# LOOK AHEAD SLEEP: STUDY DESIGN AND BASELINE CHARACTERISTICS

**DOI:** 10.1093/geroni/igad104.1698

**Published:** 2023-12-21

**Authors:** Adam Spira, Suzanne Bertisch, Haiying Chen, Jingzhong Ding, Susan Redline, Marie-Pierre St-Onge, Kathleen Hayden

**Affiliations:** Johns Hopkins Bloomberg School of Public Health, Baltimore, Maryland, United States; Brigham and Women’s Hospital, Harvard Medical School, Boston, Massachusetts, United States; Wake Forest University School of Medicine, Winston-Salem, North Carolina, United States; Wake Forest University, Winston Salem, North Carolina, United States; Harvard Medical School, Boston, Massachusetts, United States; Columbia University Irving Medical Center, New York City, New York, United States; Wake Forest University School of Medicine, Winston-Salem, North Carolina, United States

## Abstract

Older adults with obesity and type 2 diabetes (T2D) are at elevated risk for mild cognitive impairment (MCI) and dementia. Compared with younger persons, older adults are more likely to have sleep-disordered breathing (SDB), which is also common among adults with T2D and obesity and is associated with higher risk of dementia. The Look AHEAD Sleep (LA-Sleep) study investigates links of SDB and circadian rest/activity rhythms with cognitive performance, MCI, and dementia in older adults with T2D and obesity/overweight enrolled in Look AHEAD Aging (LA-A). LA-Sleep participants completed cognitive measures at baseline and were randomized to sleep measurement in the first (2022-2023) or second year (2023-2024). Sleep measures include assessments of SDB (WatchPAT), circadian rest/activity rhythms (ActiGraph) and daily sleep diary. Of those who enrolled in LA-A, 62.8% consented to LA-Sleep. To date in 203 participants have provided data from WatchPAT and 107 provided actigraphy data. Participants were a mean (±SD) age of 76.1±5.4 y, had a mean BMI of 34.2±6.7 kg/m2, 56.7% identified as female, and 75.4% were Non-Hispanic White, 12.3% Non-Hispanic Black, and 6.9% Hispanic. Approximately 90.2% had >high school education. Of participants with obesity in 2018-2020, 27.9% (41/147) had an apnea hypopnea index (AHI) of ≥15 to <30/h (moderate SDB) and 20.4% (30/147) had ≥30 events/h (severe SDB). Mean actigraphy-estimated total sleep time was 7.1±1.4 h, and mean wake after sleep onset was 62.5±31.0 min/night. Among adults with T2D and overweight/obesity, a high proportion have SDB and sleep fragmentation, potentially increasing risk for dementia.

